# Genito-Urinary and Syphilitic Diseases

**Published:** 1902-02

**Authors:** Byron H. Daggett

**Affiliations:** Buffalo, N. Y.


### PROGRESS IN MEDICAL SCIENCE.

### Genito-Urinary and Syphilitic Diseases.

CONDUCTED BY BYRON H. DAGGETT, M. D., Buffalo, N. Y.

### TECHNIC OF FIXATION OF PROLAPSED KIDNEY.

GOELET, AUGUSTIN H. (*American Medicine*, December 28, 1901,) states that prolapse of the kidney is a surgical condition, because it does not admit of cure by any other means.

In general enteroptosis, the prolapse of the kidney produces more serious consequences than the prolapse of the other abdominal organs, as the interference with its function produces more serious consequences. The relief of nephroptosis lessens the ptosis of the other organs. The operation in itself involves no risk.

A prolapsed kidney, which produces discomfort or ill health demands operation, because: (1) there is no other certain means of cure; (2) there is the tendency to progressive prolapse; (3) prolonged prolapse produces congestion and softening; and (4) it may cause serious obstruction to the function of the kidney. The patient should be as carefully prepared as for abdominal section.

*Technic of the Operation.*—The patient is placed upon the table on the well side, with the under arm drawn out and the thighs flexed. A deep wedged shaped pad is placed under the loin to push the abdomen up, increase the costo-iliac space and crowd the kidney into place. The kidney will then be within reach when the fatty capsule is exposed. Make the incision along the outer border of the erector spinæ muscle downward for about three inches parallel with the spine, beginning at the lower margin of the twelfth rib. The distance from the spine will be three and a half to four inches, according to the width of the back. Cut down to the superficial fascia, covering the muscles and cut this fascia with scissors to the length of the external wound. Separate the muscles outlaying the erector with blunt scissors and blunt hooks, making traction toward the extremities of the opening. Broad, strong retractors are inserted to retract forcibly the separated muscles. The erector spinæ can usually be retracted by including its border in the retractor on that side; if not, carefully divide the fascia with blunt scissors. This exposes the quadratus lumborum. Separate the fibers of quadratus with blunt hooks, so that the iliohypogastric nerve and vessels may be drawn aside with the retractors. Open the deep fascia and the kidney will bulge up into the wound. Include this fascia in the grasp of the retractors and stretch the wound to its fullest extent. Then draw down the fatty capsule and open it with blunt scissors from the center of the wound to its upper limit. Grasp the margins of the capsule with T forceps and draw the kidney up into the wound. If the capsule is adherent carefully separate it from the margins around and under its lower pole, nearly to the ileum. This gives a considerable area of its surface for approximation and adhesion to the muscles of the back.

It is not necessary to strip off or scarify the fibrous capsule to secure adhesion. Hold it immovably in position for a sufficient length of time and it will adhere.

There should be two fixation sutures inserted upon the lower half of the kidney, so that the upper part may be drawn up in place under the ribs.

The fixation sutures, of which there should be two, are inserted upon the lower half of the kidney so that the upper part may be drawn up into place under the ribs. A small, short, full curved needle, threaded with silkwormgut (made pliable by boiling in lysol solution) is inserted first upon the outer part of the exposed kidney surface from above downward, somewhat obliquely to the long axis of the kidney, and the suture drawn through; then it is inserted transversely and again from below upward in the same manner as in the first insertion, but on the opposite side of the exposed surface. The insertions of this suture are superficial, penetrating only beneath the fibrous capsule so as not to wound the deeper kidney structure. The free ends of this suture are caught with pressure forceps to prevent the suture from being pulled out while the second suture is being inserted.

The second suture is inserted above the first, but in a somewhat different manner, there being no transverse insertion, but an exposed loop of the suture on the outside of the fibrous capsule.

This form of suture will bear greater strain than the usual transverse suture. These sutures are passed underneath the fibrous capsule, as the kidney substance possesses no more resistance than firm butter.

After both sutures have been inserted in the manner described the free ends of the upper suture are threaded successively into a long curved perineal needle which is then inserted from within the fatty capsule at the upper angle of the wound and brought out on the skin surface on a level with the extreme limit of the upper angle of the wound about a quarter of an inch from the margin, penetrating all the structures of the back. The suture end is then detached from the needle, which is withdrawn, leaving the suture in place. The other end of the suture is carried through the structures of the back on the opposite side in a similar manner. The free ends of this upper suture are caught with pressure forceps and the ends of the second suture carried through the structures of the back from within the fatty capsule in the same manner, and are brought out a short distance below the other. The ends of this suture are also caught with pressure forceps.

The redundant fatty capsule is trimmed off on a level with the bottom of the wound on both sides, but that part below the recent location of the lower pole of the kidney is not disturbed. The wound, including the pouch within the fatty capsule below the kidney, is now flushed with warm, salt solution, carefully wiped dry, and the retractors removed. The sustaining sutures are tightened, bringing the upper pole of the kidney up under the last rib, and then tied, the upper one first, over a fold of several layers of gauze placed lengthwise at the upper angle of the incision.

The sustaining sutures are tied over a fold of gauze, so that they will not loosen by cutting into the skin.

Before closing the wound a long strip of gauze is packed under and around the lower pole of the kidney, filling the space occupied in its prolapse, thus bolstering the kidney, and lessening the strain on the suture, exciting plastic inflammation and affording drainage. The walls of the wound will fall together, although it is best to stitch the superficial fascia with two or three intercepted sutures. Approximate the margins of the skin with narrow strips of adhesive plaster. The gauze drain is removed the third or fourth day, the patient to remain in bed on the back, or only turn on the side of the operated kidney for three weeks. The retaining stitches are removed after the patient gets out of bed. For a time an abdominal support should be worn and exertion or strain avoided.

